# Clustering of neuropsychological traits of preschoolers

**DOI:** 10.1038/s41598-021-85891-2

**Published:** 2021-03-22

**Authors:** Mario Treviño, Beatriz Beltrán-Navarro, Ricardo Medina-Coss y León, Esmeralda Matute

**Affiliations:** 1 Laboratorio de Neuropsicología y Neurolingüística, Instituto de Neurociencias, Universidad de Guadalajara, Guadalajara, México; 2 Laboratorio de Plasticidad Cortical y Aprendizaje Perceptual, Instituto de Neurociencias, Universidad de Guadalajara, Guadalajara, Jalisco México; 3 Departamento de Neurociencias, Centro Universitario de Ciencias de la Salud, Universidad de Guadalajara, Guadalajara, Jalisco México

**Keywords:** Psychology, Human behaviour

## Abstract

Neuropsychological tests (targeting cognitive, linguistic, motor, and executive abilities) are grouped in neuropsychological domains that are thought to be stable through adulthood. However, this assumption does not always hold true, particularly during young children’s early developmental phase. Here, we explored how the neuropsychological profile of typical Spanish-speaking preschoolers varied and consolidated with age. We recruited 643 monolingual Latin-American children from Mexico, Colombia, and Guatemala, with ages spanning from 30 to 71 months of age, and applied a novel neuropsychological examination which combined a total of 52 tests covering five classical neuropsychological domains: receptive, expressive, attention/memory, processing, and executive functions. These tests’ scores uncovered a correlational structure across neuropsychological functions that could not be explained by chance. Notably, these correlations’ overall strength, but not their interdependence across domains, dramatically increased with age. Moreover, by applying conventional clustering techniques to classify the experimental data, we found a stable representation of two clusters of children with distinctive traits, with cultural factors contributing to this classification scheme. We also found that the tasks were well organized in a network of abilities, where nodes with highest highest interconnectedness were those that required multimodal processing. These results contribute to our understanding of children’s ‘normal’ development and could help identify how failure in particular functions forecasts the emergence of neurodevelopmental disorders. Our analytic methods might become useful to characterize individual differences and improve educational practices and interventions.

## Introduction

Historically, neuropsychological research has focused on the administration of tests to identify brain dysfunctions and learning disabilities. The traditional view, mainly derived from lesion studies, proposes that the human brain has discrete and separate modules specializing in specific functions^1^. Under this notion, it should be possible to associate a localized brain lesion with a single functional disruption. A more contemporary perspective, however, proposes that the brain can not be assumed to be modular because it is not composed of isolated parts. Indeed, evidence shows that the brain exhibits large-scale interconnectivity patterns that are incompatible with the idea of independently functioning modules. Accordingly, modern neuroscience tries to explain behavior due to complex interactions across multiple brain areas^2^. For example, executive functions, which depend on the prefrontal cortex and subcortical systems, are generally described as multidimensional constructs consisting of several interrelated sub-functions^3–5^. These sub-functions include volition, goal selection, planning, inhibition, cognitive flexibility, working memory, and evaluating the results of actions, among others. Neuroconstructivist and developmental approaches also view the brain as a dynamic self-organized system with internal (physiological, genetic) and external (context) factors that shape it’s maturation, also with patterns of activation becoming specialized through this process^1^. Therefore, almost every aspect of development turns out to be dynamic and interactive^6^.

Many studies have shown how neuropsychological functions unfold from infancy to adolescence, within different hierarchical modules^3,7–9^. These modules develop at different rates, starting on subcortical areas that allow arousal, followed by the maturation of regions participating in motor and sensory functions. After that, circuits involved in motor coordination, spatial orientation, and language develop. Similarly, the development of integrative and multimodal functions associated with tertiary sensorial areas gives rise to abilities such as reading, writing, and arithmetic skills. The prefrontal cortex is the last area to mature: it participates in executive functions necessary for goal-directed behavior^10^. Consequently, some impairments in any of these functions/regions are associated with a range of disorders and severities according to the neurodevelopmental phase when they ocurr.

Trying to understand the brain as a complex system gives rise to novel questions. How do cognitive functions change and interact over developmental time, and what are the internal and external elements that affect such cognitive development? Consider, for instance, the development of language. Here, the maturation of brain areas involved in motor control (secondary motor areas) allows the production of phonemes. Isolated words and sentences are then produced in coordination with secondary motor areas. These abilities are the precursors of storytelling, which requires a complex interaction between memory capacity and the presence of semantic and morphosyntactic components^11^. Reading and writing appear around 6 years of age as multimodal functions, where complex metalinguistic processes arise. Finally, the maturation of the prefrontal lobes unfolds as a multistage process, with distinctive abilities improving in different ways and at different rates until reaching maturity in adulthood^12^. This maturation process allows planning skills, working memory, self-reflexive processes (crucial for written expression), argumentation, and critical thinking.

All in all, although the brain completes its development until adulthood, the neurodevelopmental processes during childhood are markedly different across ages. For example, there is a high processing variability across multiple neuropsychological domains in children below 6 years of age, particularly in executive functions. These early developmental phases are highly relevant because they impact numerous functional areas, such as school and social performance. Furthermore, the immaturity of neuropsychological functions in young children implies that they can be vulnerable to cerebral insults. For all these reasons, it is crucial to characterize whether and how relevant neuropsychological functions change during the early stages of child development.

Here, we explored how the neuropsychological profile of typical monolingual Spanish-speaking Latin-American preschoolers varied and consolidated with age. Our main aims were to: (1) track the neuropsychological development in these preschoolers, (2) determine how domestic and educational factors were related to their neuropsychological profiles, and (3) explore whether and how these abilities were organized and interrelated. We employed a battery of 52 tests, grouped into five neuropsychological functions: receptive, expressive, attention/memory, processing, and executive ones. This battery was developed following the conceptual framework of Lezak et al.^5^, and standardized for Spanish-speaking Latin-American children^13^. Experienced psychologists carried out testing, and the children were organized into seven age groups, ranging from 30 to 71 months of age. Our analytic results revealed well-orchestrated changes across age groups that were influenced by educational and other socio-cultural variables. Interestingly, even though our participants were typical middle-class preschoolers, they could be classified into two well-differentiated clusters. Our analysis also revealed that the neuropsychological abilities conformed a network of well-interconnected nodes. Within this network, the tests belonging to expressive, processing, and executive functions exhibited the highest interconnectedness. Our results and methods serve to understand the development of neuropsychological functions and how individual differences emerge; they may be useful for educational practices and interventions.

## Results

### Classification of Latin-American children across neuropsychological functions

**Figure 1:**
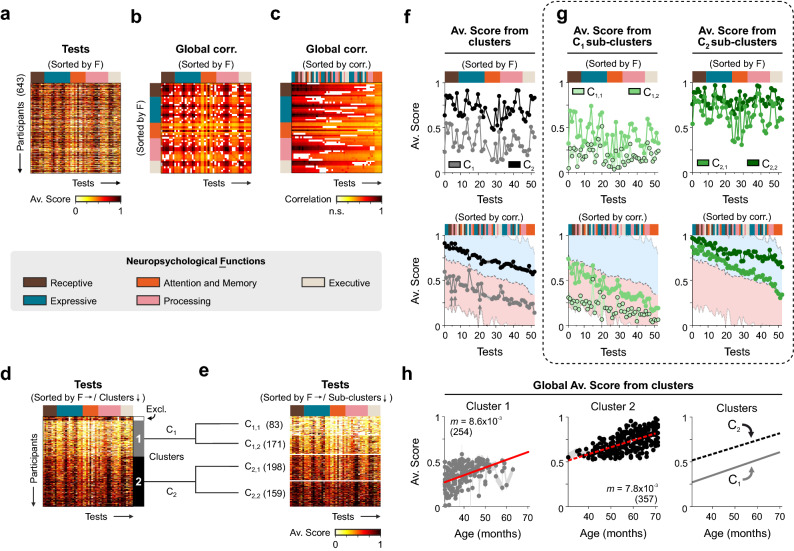
Classifying Latin-American children’s performance across distinct tasks and neuropsychological functions. (**a**) Scores from a battery of 52 neuropsychological tests performed on 643 Latin-American children. Grouping of tests into receptive (walnut brown), expressive (mosaic blue), attention/memory (orange), processing (flamingo pink), and executive (cloud cream yellow) functions (color bar on the bottom of the heatmap). (**b**) Peak global cross-correlations across tasks sorted by functions or (**c**) by the summed correlations across tasks for the entire data set. Filled pixels represent values that cannot be explained by chance (*P* < 0.05). (**d**) Classification of children into two main clusters (C_1_ and C_2_). (**e**) Further partition of the two main clusters into two sub-clusters each (C_1,1_, C_1,2_, C_2,1_, and C_2,2_). (**f**) Average scores ± S.E.M. for the groups of children from the main clusters sorted by neuropsychological functions (top) or by the summed correlations (bottom). Gray arrows point to three tests that exhibited average values below those expected if they followed the trend of scores sorted by correlation (test #5: Left hand recognition; #7: Following oral instructions; #21: Naming body parts). (**g**) Average scores from each sub-cluster sorted by neuropsychological functions and by the summed correlations (same arrangement as in **f**). Shaded areas in panels F and G depict the regions covering 1.5 standard deviations away from the mean of the population, which is shown as a grey dotted line. (**h**) Global scores (i.e., the average of all tests) as a function of age for C_1_ and C_2_. Linear fits display similar slopes but different intercepts for each cluster. Number of participants in parentheses.

Typically, examiners use neuropsychological tests (covering cognitive, linguistic, motor, and executive domains) to explore discrete responses associated with particular functions. First, we wondered if such apparently discrete abilities (responses) do not occur in isolation but, instead, they contribute to more complex skills. For this, we characterized the distribution of neurocognitive abilities in Latin-American children from 30 to 71 months of age. We applied a battery of 52 tests (Supplementary Table 1) covering receptive (8 tests), expressive (15), attention/memory (9), processing (13), and executive functions (7), on a total of 643 typical children (Supplementary Table 2). We merged the data from female and male children because there were no gender differences when comparing their averaged scores (two-tailed Wilcoxon rank-sum test; *P* = 0.15). These tests’ normalized scores revealed a strong task-dependency, yet with a high heterogeneity across children (Fig. 1a). We thus wondered how the scores obtained from the different tests were related to domains within the same and other neuropsychological functions. Intuitively, one would expect similar scores among tests belonging to the same neuropsychological function. We performed a correlation analysis across all tasks using the entire database and tested the observed correlations’ significance. We did this by creating surrogate comparison data (*n* = 1000) by shuffling each participant’s scores across the 52 tests. In Fig. 1b, we illustrate the cross-correlation matrix derived from this analysis with significant relationships marked as colored squares and non-significant (n.s.) values appearing as empty squares (i.e.,* P *≥ 0.05). This matrix reveals a quite heterogeneous structure with strong and weak relationships among tests that belong to practically all functions (represented with different color bars to the left and above the correlation matrix). Interestingly, tests from attention/memory functions (in orange) exhibited smaller correlations than the rest of the tests (one-tailed Wilcoxon rank-sum test; *P* ≤ 0.027 for all cases). To explore alternative relational schemes among tests, we re-organized the correlation matrix by sorting its columns by the summed correlations across all tests (from highest to lowest; Fig. 1c). The resulting heterogeneous distribution of functions (to which the sorted tests belonged) reveals intermingled functions for strongly correlated tests (illustrated in the color bar on top of the correlation matrix).

Next, we wondered whether the high heterogeneity in the children’s scores across tasks could reflect particular sub-groups of children with specific skills within the sampled population. We applied a nearest neighbor classification technique using the Euclidean distance as a measure for cluster center initialization. Using the Calinski-Harabasz and the Davies-Bouldin criteria (Supplementary Fig. 1a–e), we found that the original data set could be organized into two main clusters (C_1_ and C_2_; Fig. 1d). Using the same optimization criteria, we found that these two clusters could be further subdivided into two additional sub-clusters each (Fig. 1e and Supplementary Fig. 1f,g). The two main clusters differed in that cluster 1 (C_1_) was associated with ~ 47% lower average scores across all tasks compared to cluster 2 (C_2_; scores C_1_: 34.15 ± 0.84%, *n* = 254; scores C_2_: 71.44 ± 0.57%, *n* = 357; Kruskall–Wallis multi-comparison test with Bonferroni *posthoc* correction; *P* < 0.0001 for all tests; Fig. 1f). Practically all functions were similarly reduced in C_1_ relative to C_2_ (i.e., they were scaled-down). Only three tests exhibited average values below those expected if they followed the trend of scores sorted by correlation (Tests #5: Left-hand recognition; #7: Following oral instructions; #21: Naming body parts; depicted with gray arrows in Fig. 1f). We also found a similar pattern of distinctive average scores across all tests for the corresponding sub-clusters (C_1,1_: 18.65 ± 1.10%, *n* = 83; C_1,2_: 41.68 ± 0.51%, *n* = 171; C_2,1_: 63.36 ± 0.38%, *n* = 198; C_2,2_: 81.49 ± 0.49%, *n* = 159; KW test with Bonferroni *posthoc* correction; *P* < 0.0001; Fig. 1g). Suggesting a graded transition, the correlation in average scores across all these four classifications revealed stronger similarities between contiguous clusters (not illustrated).

Finally, we searched for potential differences across ages in the neuropsychological functions between the two main clusters. We plotted the average scores from all tests against the age of the children from each group. Interestingly, these two clusters had different intercepts (*b*) but similar slopes (*m*; C_1_: *m* = (8.6 ± 0.5) × 10^–3^, *b* = (1.2 ± 0.5) × 10^–3^; *P* < 0.001; C_2_: *m* = (7.8 ± 0.4) × 10^–3^, *b* = (274.6 ± 2.4) × 10^–3^; Kruskall–Wallis test with Bonferroni *posthoc* correction; *P* < 0.001; Fig. 1h). This result suggests that both clusters exhibited similar improvements with age (as inferred by the slopes of these regressions). Still, each of them was associated with a different performance level. The different intercepts could be explained by external (*v.gr.,* environmental) or internal (*v.gr.,* genetic) conditions related to each group.

Our overall sample of Latin-American children was composed of 76.92% Mexican (*n* = 470), 14.24% Colombian (*n* = 87), and 8.84% Guatemalan (*n* = 54) children (Supplementary Fig. 1h). The difference in the number of participants from each country creates the potential concern that the optimal number of clusters that we found for the entire dataset could be sensitive to educational or cultural differences among these nationalities. We adjusted and compared the linear regressions to the children’s global performance by their nationalities (i.e., the average score from all the tests/participants) as a function of age (Supplementary Fig. 1i). We made size-matched linear regressions from sub-sampled groups of children (20–90% with 10% increments, 1000 iterations/category) and found relevant differences in the slopes and intercepts among groups (Supplementary Fig. 1j). More specifically, Mexican children displayed slightly faster increases in their neuropsychological functions (i.e., higher slope) than Colombian and Guatemalan children, yet, the Colombian children had a higher intercept than the other two groups (Mexico: *m* = (1.62 ± 0.05) × 10^–2^, *b* = -(22.42 ± 2.77) × 10^–2^; Colombia: *m* = (1.43 ± 0.14) × 10^–2^, *b* = -(7.88 ± 6.54) × 10^–2^; Guatemala: *m* = (1.38 ± 0.20) × 10^–2^, *b* = -(20.67 ± 11.79) × 10^–2^; Kruskall–Wallis multi-comparison test with Bonferroni *posthoc* correction; *P* < 0.0001, for all cases; Supplementary Fig. 1k). We confirmed these differences by re-calculating the slopes and intercepts from size-matched groups (Supplementary Fig. 1l). Given these results, we then tested whether nationality differences could compromise the entire dataset’s sub-clustering structure. We repeated the clustering analysis on the Mexican children only and found a similar classification structure as the one observed for the whole population (of Latin-American children). Namely, on an initial partition, we confirmed the existence of two main clusters, which could be further divided into two sub-clusters each (using Calinski–Harabasz and the Davies–Bouldin criteria; Supplementary Fig. 1m,n). These results confirm the notion that the scores obtained from Latin-American children solving our 52 tests can be organized into four sub-clusters, which exhibit a graded transition in performance across tests.

### Development of neuropsychological functions

**Figure 2:**
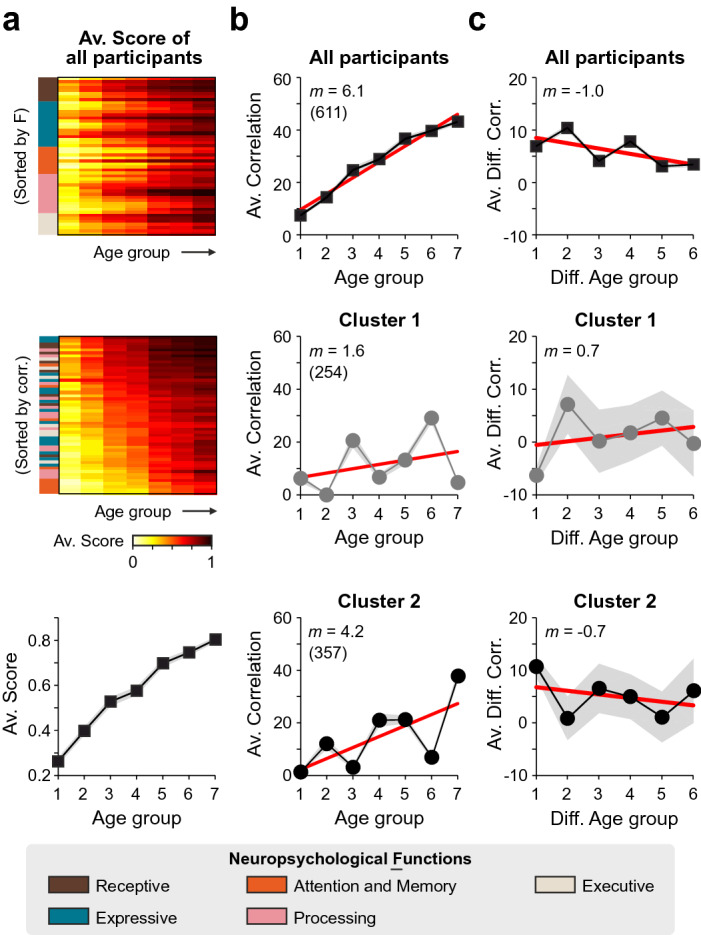
Differential developmental control of neuropsychological functions between identified clusters. (**a**) Average scores as a function of age group sorted by functions (top) or by increasing correlations (middle). The lower panel shows the average scores from all 52 tests. Averaged (**b**) and averaged differences (among contiguous age groups; **c**) in cross-correlations for all tests as a function of the age group for all participants (top), and participants from cluster 1 (center), and cluster 2 (bottom). Red lines depict linear fits with corresponding slopes displayed on the top of each panel. Number of participants in parentheses.

Neuropsychological functions develop from infancy into adulthood^14,15^. We wondered if the children’s scores for all tests improved similarly with age since we considered high performance as an indirect developmental measure. We used a colormap to illustrate the age-related increases across neuropsychological functions and found distinctive neurodevelopmental profiles (colormaps in Fig. 2a). Since the group averaged scores from our tests presented such a strong dependency on age (lower panel in Fig. 2a), we wondered whether the correlation structure across all tasks could involve a strong neurodevelopmental control. For this, we computed the correlation matrices across tasks from children belonging to each of the seven age groups (ranges: 30–35, 36–41, 42–47, 48–53, 54–59, 60–65, and 66–71 months), and confirmed that the overall correlations across tests increased and became more selective with age (Repeated Measures One-Way ANOVA test, *F* = 29.32, *P* = 0.001; Fig. 2b, Supplementary Fig. 2a). To get an overall estimation of how these correlations varied with age, we calculated the average correlation across all tests as a function of the age group for members from clusters C_1_ (middle panel of Fig. 2b) and C_2_ (lower panel of Fig. 2b). Tests correlations increased with age for both groups (RM One-Way ANOVA test, C_1_: *F* = 52,207.75, *P* < 0.001; C_2_: *F* = 33,168.66, *P* < 0.001), yet children belonging to C_2_ had, on average, a ~ 2.5 higher performance than children from C_1_ (middle and lower panels in Fig. 2b; C_1_: *m* = 1.6 ± 0.1, *b* = 5.04 ± 0.01; C_2_: *m* = 4.2 ± 0.1, *b* = − 1.92 ± 0.1; KW-test, *P* < 0.005 for slopes and intercepts).

To estimate if the within-group differences decreased with age (*v.gr.* as one would expect for correlations reaching an asymptote), we calculated the first derivative of the average correlation by taking the difference between average correlations across matrices from contiguous age groups. We found no clear reduction in these numbers through age groups (RM One-Way ANOVA test, *F* = 0.47, *P *≥ 0.05, for both cases; Fig. 2c and Supplementary Fig. 2d), suggesting an un-finished developmental process. These analyses reveal that children from C_2_ exhibited a higher increase in their average correlation profile than children from C_1_. Thus, in principle, children from C_2_ could be maturing their cognitive functions earlier than children from C_1_. Finally, we also explored if the cluster centroids (and/or the difference between them) varied between age groups. We used a colormap to illustrate the values associated with the cluster centroids. We found, as expected, higher average scores in older age groups (Supplementary Fig. 3a,b). However, the absolute difference between cluster centroids revealed widespread effects across neuropsychological functions with no evident age-related change (Supplementary Fig. 3c). This result is consistent with the fact that both groups exhibited similar changes in their neuropsychological functions through age (Fig. 1h).

### Domestic and educational factors linked to neuropsychological classification

**Figure 3:**
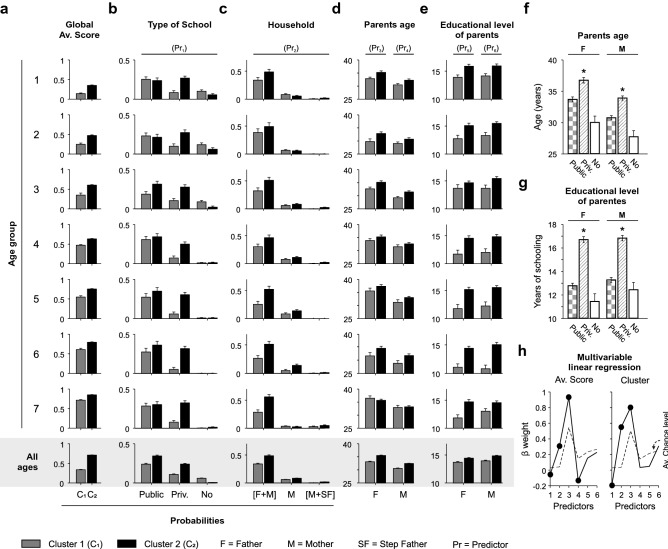
Influence of domestic and educational factors on cluster classification. (**a**) Global scores (i.e., the average from all tests) for the participants from cluster 1 (C_1_, in gray) and cluster 2 (C_2_, in black). (**b**) Probabilities that children from C_1_ and C_2_ went to public, private, or no-school (Predictor 1: Pr_1_). (**c**) Probabilities that children from C_1_ and C_2_ lived with both parents, only with the mother or with mother and stepfather (Pr_2_). (**d**) Average age of fathers (left, Pr_3_) and mothers (right, Pr_4_) of children from C_1_ and C_2_. (**e**) Average years of schooling of fathers (left, Pr_5_) and mothers (right, Pr_6_) of children belonging to C_1_ and C_2_. Average age (**f**) and average years of schooling (**g**) of fathers (left) and mothers (right) of children attending public, private, or no-school. (**h**) Multivariate regression model exploring how the six predictors (Pr_1–6_) influenced cluster assignment (significant regression weights marked with filled dots).

Biological (*v.gr.* weeks of gestation, birth weight, head circumference^16,17^, and environmental (*v.gr.* Family background, marital histories, socio-economical levels^12,18–20^) factors influence children’s neuropsychological development. Since we controlled prenatal factors in our sample (*v.gr.* weeks of gestation; see “Methods”), we next explored whether and how some domestic (household as well as parents age, and educational level) and educational (type of school) factors were related to the observed neuropsychological functions and corresponding classification schemes of the children. We calculated the average normalized scores (i.e., the average of the scores obtained from the 52 tests) associated with cluster 1 (C_1_, in gray) and cluster 2 (C_2_, in black) from children belonging to the different age groups (ranges: 30–35, 36–41, 42–47, 48–53, 54–59, 60–65 and 66–71 months; Fig. 3a). We then calculated the probability that the children belonging to these two clusters attended either a public, private or no school at all (Fig. 3b). Notably, children belonging to C_2_ (i.e., w. higher scores) were ~ 120% (all ages) more prevalent in private schools than children from C_1_ (i.e., w. lower scores).

Next, we investigated if household conditions, like children living with both parents, mother only, or with mother and stepfather, influenced their classification into their respective clusters (Fig. 3c). Children from C_2_ were ~ 43% more likely than those from C_1_ to belong to a family with both parents. Similarly, we found that the average age of the parents (Fig. 3d) and their average educational level (Fig. 3e) influenced the classification groups, where older parents with a higher educational level were associated with C_2_ (Supplementary Tables 2 and 3).

At first sight, the fact that children attending private schools were more likely to belong to C_2_ could be explained by differences in the education programs provided by private *vs.* public schools. However, such a relationship could also be a consequence of other domestic factors such as parents’ age or their educational level. We explored our database and found that parents of children attending private schools were older, on average, than those of children attending a public or no school (age father: *F* = 50.75, *P* < 0.001; age mother: *F* = 58.51, *P* < 0.001; Fig. 3f). Similarly, the average educational level of the parents of children attending private schools was higher than that of parents of children attending public or no school (years of schooling father: *F* = 119.85, *P* < 0.001; years of schooling mother: *F* = 112.37, *P* < 0.001; Fig. 3g). Therefore, the score differences in children attending public and private schools depended on factors existing outside the school^21^. Additionally, using a multivariable linear regression model, we explored how specific predictors (Pr_1_: type of school, Pr_2_: household, Pr_3_: father’s age, Pr_4_: mother’s age, Pr_5_: educational level of the father, Pr_6_: educational level of the mother) explained: (1) the global scores of the children (left panel in Fig. 3h), and (2) the classification clusters of the children (right panel in Fig. 3h). In Fig. 3h, significant coefficients for predictors (Pr_1_–Pr_6_) compared against surrogates (obtained by permuting the predictors) are illustrated with filled dots. These results reveal that type of school, household, parents’ educational level, and parents’ age influence the scores and classification schemes of the children’s neuropsychological functions.

### Integration of neuropsychological functions through development

**Figure 4:**
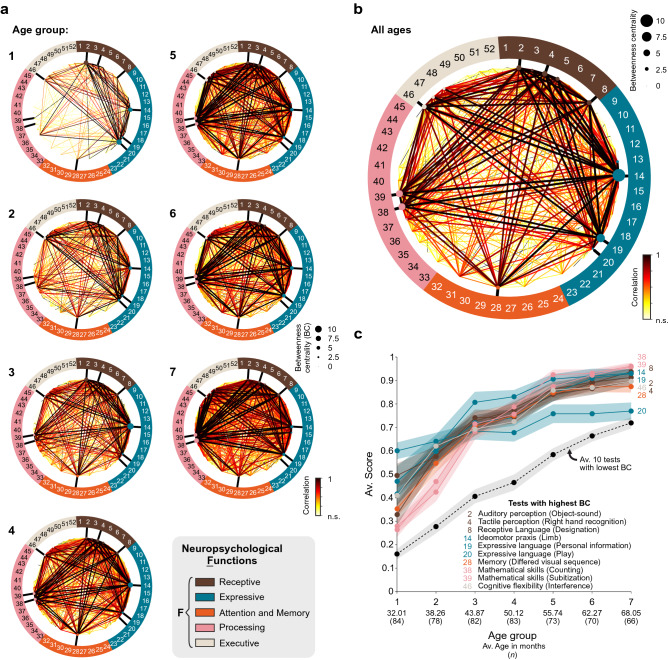
Consolidation/integration of critical neuropsychological functions through development. Network visualization of the cross-correlations across the 52 neuropsychological tasks using the scores from children belonging to each age group (**a**) or from all ages (**b**). Tasks appear as nodes radially arranged along a circumference covering the following domains: receptive (walnut brown), expressive (mosaic blue), attention/memory (orange), processing (flamingo pink), and executive (cloud cream-yellow) functions. The colors of the lines and their thickness represent the cross-correlation between pairs of tasks, whereas the area of each node is proportional to the betweenness centrality (BC) for each task. (**c**) Developmental profile for the ten tasks with the highest BC compared against the average of ten tasks with the lowest BC (dotted line).

Colormaps illustrating correlation matrices or neuropsychological functions help establish paired comparisons across tests (*v.gr.,* Fig. 1b). However, tests that concentrate high correlations cannot be easily identified using these graphs. Therefore, we implemented a ‘network representation’ where we distributed the 52 tests as discrete nodes along a circumference, with the lines’ thickness and colors connecting these dots representing the cross-correlation level between each pair of nodes (tests). These plots were made with data from each age group (Fig. 4a) and the entire dataset, including all ages (Fig. 4b; see Supplementary Table 1). Besides, we implemented centrality measures from network theory^22^ to explore each test’s relevance within this network of functions. Many of these metrics are based on the idea that well-interconnected nodes are likely to facilitate global, intermodular integration. We thus calculated the betweenness centrality (BC) of each node to identify relevant connections with other tests within the battery. We represented the BC of the tests as the area of the circular nodes in Fig. 4a,b. Interestingly, the ten tests with the highest BC exhibited a faster increase across age groups than the average of ten tests with the lowest BC (dotted line in Fig. 4c). This result suggests that expressive and receptive functions establish earlier their foundations with abilities that promote the development of other skills. Interestingly, it is after 4 years of age that more complex abilities, such as math skills and cognitive flexibility, show complex connections.

**Figure 5:**
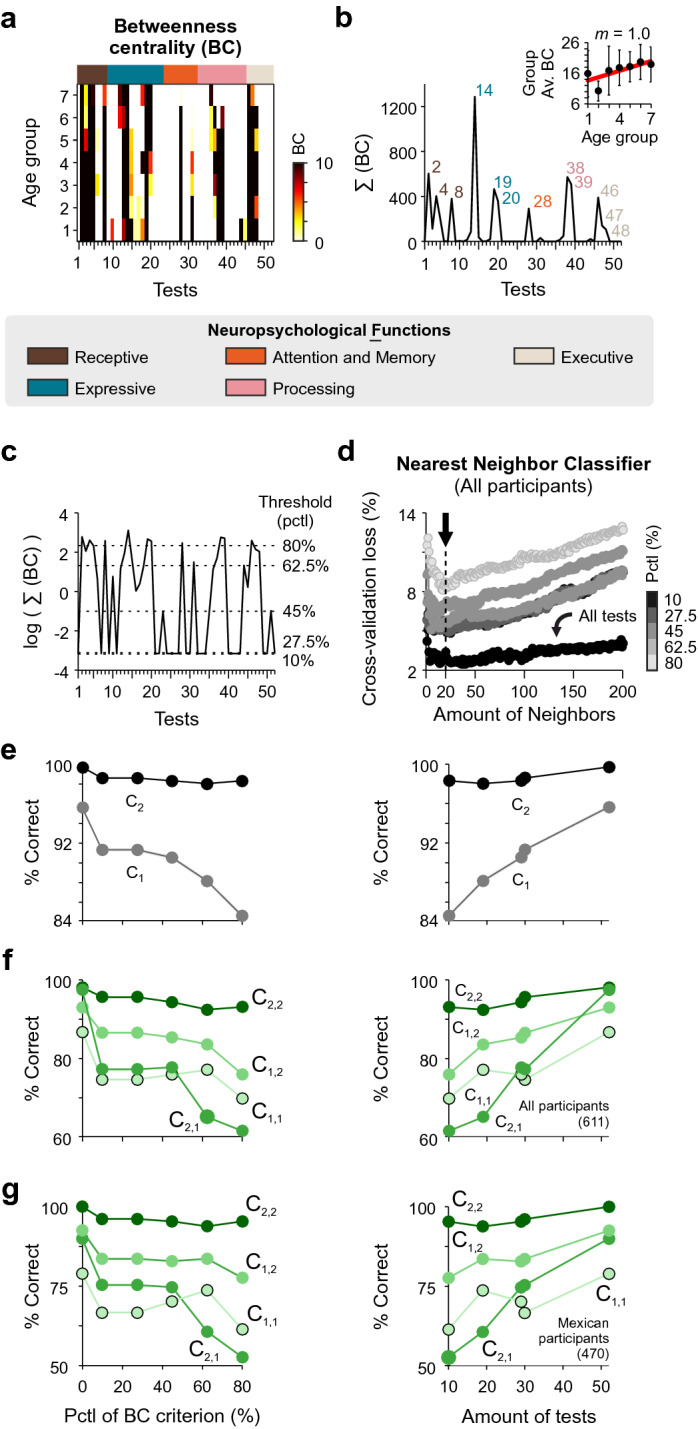
Supervised classification with centrality measures. (**a**) Colormap illustrating the betweenness centrality (BC) for each task as a function of age group. Binary BC was computed by thresholding each normalized correlation matrix against 0.5. Colorbar on the right. Summed BC (across ages) for the different tests in linear (**b**) and logarithmic (**c**) scales, respectively. (**d**) Cross-validation loss of a k-nearest neighbor (KNN) classifier as a function of the number of neighbors. Predictors consisted of the different tests associated with the different threshold values for BC. Optimal number of neighbors is around 20. (**e–g**) Performance of the KNN classifier as a function of the %BC criterion (left column), and as a function of the number of tests that correspond to that BC criterion (right column). The panels display the performance in classifying the children from the two main clusters for the entire dataset (**e**), the four sub-clusters for the entire group (**f**), and the four sub-clusters for the Mexican children only (**g**). Number of participants in parentheses.

The BC of the tests exhibited distinctive values (Fig. 5a) and consolidated across age groups (inset in Fig. 5b). To get an overview of the centrality for all ages, we calculated the summed BC for all tests across all age groups (Σ(BC)) and plotted the resulting data in linear (Fig. 5b) and logarithmic (Fig. 5c) scales. Using increasing thresholds given by different percentiles from the summed BC distribution, we created five groups of tests that exhibited increasing centrality values for all ages (Fig. 5c). Lower thresholds implied a more flexible rule with more tests having an equal or lower BC than the thresholded value (Fig. 5c). As a reference, the ten tests with the highest BC measures are listed in Fig. 4c.

Biometric systems serve to identify individuals by using physiological and behavioral predictors. We hypothesized that scores from tests with high BC could be employed to identify the children^23^. We used the five groups of tests with different BC levels (in scales of gray) as predictors and combined them with supervised machine learning algorithms to predict the cluster to which the children belonged (Fig. 5d). We first cross-validated a k-nearest neighbor (KNN) classifier. We found that the classification improved with the number of tests employed as predictors and that ~ 20 neighbors optimized the classification procedure (black arrow in Fig. 5d). Next, we measured the performance of the trained classifier in identifying children from each cluster. Notably, identification of the best performers (i.e., children belonging to C_2_) was more efficient and required fewer tests than for children from C_1_ (right panel in Fig. 5e; KW-test, *P* < 0.0001 for all cases). Also, as expected, adding tests to the battery of predictors increased the classifier’s prediction capacity when dealing with children from both C_1_ and C_2_ (right panel in Fig. 5e; RM One-Way ANOVA test, *P *≥ 0.001 for both cases).

Next, we used the same 20-neighbor KNN-classifier to challenge the prediction of the children’s belonging to one of the four sub-clusters. We found similar tendencies as before: higher predictiveness required using fewer tests for higher performers (Fig. 5f). We confirmed these observations when applying the same KNN-classifier to Mexican children only (Fig. 5g). Altogether, these results illustrate how neuropsychological scores served to identify the children, where higher scores led to a more accurate classification. Therefore, neuropsychological tests’ scores can be used as behavioral ‘fingerprints’ to identify participants with a high level of certainty.

## Discussion

We explored age-related changes across neuropsychological functions from a large sample of young Latin-American children in the age range of 2.5–6 years (30–71 months of age). We used a battery of 52 standardized tests, designed for Spanish-speaking children^13^, to explore the developmental profile of receptive, expressive, attention/memory, processing, and executive functions.

We identified age-related gains in scores across all tasks, which reflect an organized development of neuropsychological functions, as has been reported^4,24–28^. We applied a cluster analysis to our data and identified two main clusters (C_1_ and C_2_) associated with a specific configuration that suggests that not all functions developed equally in all children. Indeed, our results showed distinctive developmental trajectories of neuropsychological functions in preschoolers, as others have suggested^3^. Although similar age-related increases were observed for both cluster groups (i.e., similar slopes), C_1_ lagged behind C_2_. Importantly, C_1_ was not composed of learning disabled children since we controlled biological factors related to neurodevelopmental disorders, and it involved a large proportion of the entire sample (42%). However, C_1_ children exhibited weaker performance in all tests, and particularly lower in left-hand recognition, instructions, and naming tests. These tests involve inter-modal information transfer, receptive language, and vocabulary, respectively. Differences in right/left hand recognition tests are usually linked to functional variabilities between right/left cerebral hemispheres. In sum, such cluster differences might be relevant to establish criteria to identify children with low performance in multiple neuropsychological domains and a potential developmental delay.

Nowadays, accurate assessment of neuropsychological functions in children requires collecting cognitive, linguistic, motor, executive, emotional, and behavioral information together with the social environment, school, and household conditions. Increasing evidence suggests that environmental factors influence neuropsychological functions. This influence is evident when considering children from unstable families and with disruptive family events^29^, and early abilities can predict later educational achievements^30–33^. Thus, human development has to be studied within a context^34^, even if it’s considered a ‘homogeneous’ one. Because young children tend to spend more time with their families, we explored such variables’ influence on the preschoolers’ neuropsychological development. In Latin-America, preschool attendance is not yet universal, and most of the children attend public preschools. Therefore, to reflect the characteristics of preschool attendance in a Spanish-language ambiance, we evaluated more children from public than from private schools, in addition to a small percentage of children that did not attend any preschool/day-care. The family composition and parents’ educational level exhibited a wide dispersion. We confirmed that the children’s scores and classification clusters were sensitive to these environmental variables, particularly those related to parents’ education^5,35–37^. Children belonging to the high-performance cluster (C_2_) were more prevalent in private schools than children from the other cluster (C_1_), and they were also more likely to belong to a family with both parents present. Public schools in Latin-America tend to have more children per classroom, with a single teacher giving classes from different subjects and a limited budget to acquire teaching material. However, both educational systems do also have plenty of similarities. For example, both types of schools have mentoring schemes that adjust the educational programs to the child’s capacities. Similarly, they both inform and orient the parents regarding the child’s special needs and provide a psychoeducational assessment. Public schools also have external education units where children with special needs are partially attended.

At first glance, one could be prone to think that the education provided by private schools in Latin American countries stimulates cognitive development to a higher degree than public schools do. Nonetheless, given that many of our sample children just started school, this assumption might not be valid. We found that children from private schools were more likely to belong to a family with both parents present. Also, their parents had a higher level of education and were older than those of children attending public schools. Thus, distinctive familial contexts were associated with low- (C_1_) and high- (C_2_) performance clusters in several ways: family composition and parents’ age and education level.

It has been reported that parent’s level of education co-varies with the children’s cognitive performance^3,21,38,39^. In general, parents with high educational profiles tend to create enriched environments for their children^40–42^, and use richer vocabulary^43,44^, leading to faster language development, higher school attendance, and better performance in cognitive tests^45–47^. Other environmental factors might also contribute to children’s development, such as financial resources, parental styles, the system of values, among others^48^. Our results suggest that the higher number of C_2_ children in private schools derives from more stable families, consolidated over more years, where parents have better jobs and, therefore, exhibit a higher socio-economical status, allowing them to afford a private education for their children. The family/school ambiance probably improves the children’s development. Indeed, children’s interaction with a variety of factors that promote or inhibit neuropsychological development starts well before they begin school^21^.

We found a general increase in performance over time as the children matured. Many of these improvements could be associated with a response to repeated experience and learning^49^. However, we found that not all functions developed at the same time. To quantify the importance of individual ‘network nodes’ during this process, we implemented a developmental correlation analysis that included centrality measures^22^. We found that tests with the highest betweenness-centrality (BC) values were similar for all age groups and belonged to the five neuropsychological functions. The ten tests with the highest BC measures involved language (tests 8, 19, 20), auditory (test 2) and haptic (test 4) perception, praxis (test 14), visual memory (test 28), mathematical skills (tests 38, 39), and cognitive flexibility (test 46). In contrast, the ten tests with the lowest BC values did not involve processing and executive functions.

Strong correlation among tests might reflect that they share common underlying input variables. However, the lack of such correlations may not necessarily reflect independence between neuropsychological functions^3^. The interdependence among all these abilities is relevant because basic-level deficits (e.g., attention) could affect and limit the emergence of other abilities (e.g., language). Indeed, neuropsychological functions’ development depends on frontal functioning orchestrated with other interacting systems’ maturation, including attention, memory, language, and emotions^12^. Our results show heterogeneous correlations among tasks, supporting a ‘multidimensional’ notion for neuropsychological functions. They also suggest that abilities with high centrality could help establish the foundations for other skills to emerge. Alternatively: high centrality abilities could require many other functions to consolidate. Both perspectives imply that brain regions interact and facilitate functional integration of more advanced skills.

Many researchers suggest that a sequence in the mastery of skills required for successful development through childhood, coinciding with the maturation of the cerebral cortex^4^. For example, previous works have shown how inhibitory control’s development precedes maturation of other more elaborate functions, such as selective attention^3^. Our analysis revealed that, although functions were interrelated, their distinctive developmental trajectories were well separated already from the youngest age groups^3^. More (cumulative) changes probably occur during subsequent years. Some studies suggest a progression of executive skills in which proficiency is still not achieved by 12 years of age^50,51^.

Neuropsychological functions are crucial to adjust and adapt to changing environmental demands. Delays in neuropsychological functions’ maturation can affect how children interact with the environment, leading to other problems in cognitive, academic, and social dimensions^4,52^. In this study, we controlled some aspects of the biological background, excluding from our sample children with genetic aberrations and early CNS insults that would skew their development. Still, we found clear distinctive developmental trajectories linked to domestic and educational factors. This finding supports the idea that the environment shapes the way internal (genetic) variables are expressed. Prenatal and postnatal experiences determined the child’s development rendering a wide range of neuropsychological profiles evident as early as 30 months of age. Our approach highlights the importance of tracking developmental trajectories with an apparent heterogeneity across Latin-American countries.

Dynamic variations in the child-environment dyad give rise to distinctive phenotypes. Neurodevelopmental disorders tend to originate from basic-level deficits that have ‘cascading’ effects on other domains of the developing system, including cognition, executive functions, language, social functions, motor functions, and behavior control. Several diagnoses fall within these categories, including learning and intellectual disabilities, attention deficit hyperactivity disorder (ADHD), and communication disorders. Our results highlight the fact that identification and clasiffication of the children with low scores requieres a comprehensive battery of tests. Such instruments enable reliable diagnostics. Thus, an exciting area for future research will explore the implications of the deficits and developmental delays of neuropsychological functions associated with common neurodevelopmental disorders^53^. It will be crucial to describe the differences at multiple levels (genetic, neural, cognitive, environmental) of typically developing children and children with developmental delays and how these differences increase/decrease over time^1^.

Classification algorithms using large multidimensional biological datasets can be used for prognosis (predicting risk from neuropsychological markers), diagnosis, and therapeutic intervention. These methods can be useful for identifying low performing children and then testing the effectiveness of interventions. Proper classification would provide schools information about children’s development and might help to diagnose possible neuropsychological disorders. Moreover, classification schemes could guide patient care and treatment of neurodevelopmental disorders, or brain-injured children with cognitive, linguistic, motor, executive, and behavioral therapies based on neuropsychological knowledge. In this context, one of our interests is to provide Latin-American schools, clinicians, and scientists with specific data to guide them when assessing preschoolers’ development.

A common critique of neuropsychological tests is that their scores are imperfect indicators of latent variables. As mentioned before, neuropsychological functions are complex and multidimensional constructs and, therefore, interpretation of test scores is difficult to make^3^. Furthermore, the quantification of neuropsychological functions in children is more challenging than in adults. First, assessing these functions only constitutes a snapshot at a particular time. Second, the tests are limited and may not be sensitive to more subtle developmental changes. Another limitation of our work is that we did not perform a longitudinal study, meaning that we did not map the developmental trajectories for individual children. Longitudinal studies of children could provide information regarding the intra-individual rate and extent of development of such functions. Such approximations could help classify individual differences. Furthermore, it would also be crucial to explore the degree of plasticity in the neural networks that mediate neuropsychological functions.

Instead of considering a single compromised neuropsychological function, this work explored and described a broader neuropsychological profile of preschoolers. Using clustering analysis at multiple levels, we assessed the children’s performance for each task, the developmental stage of each function, and the interrelations between neuropsychological domains. We also explored the interplay between cognition and experience. Our results highlight the fact that not only biological and environmental factors drive and support cognitive development: there is a third factor related to how children build their knowledge, namely, how their own cognitive characteristics enable them to process the stimuli provided by their environment.

## Methods

### Participants

Data acquisition consisted of applying a Neuropsychological battery of tests (Supplementary Table 1) on a total of 643 typical (born at ≥ 37 weeks of gestation; birth weight between 2500 and 4000 g; no reports of prenatal, perinatal, or postnatal complications, or TBI, without a neurodevelopmental disorder diagnostic) Latin-American children (315 boys, 328 girls) ranging from 30 to 71 months of age. The database involved scores from 473 Mexican, 115 Colombian, and 55 Guatemalan children with similar socioeconomic backgrounds.

### Tests

The evaluation instruments were based on procedures developed in cognitive neuroscience and were standardized for Spanish-speaking preschoolers^13^. The 52 subtests belong to twenty domains grouped into five classical cognitive functions^5,54^: (1) *receptive functions* (domains: visual, auditory, haptic perception; they involve the processing of sensory information and receptive language), (2) *expressive functions* (domains: fine motor, ideomotor praxis, constructional abilities, graphic skills, gross motor, and expressive language), (3) *attention/memory functions* (domains: coding, delayed recall, attention), (4) *processing functions* (domains: reasoning, math, and pre-reading skills), and (5) *executive functions* (domains: cognitive flexibility/shifting, self regulation working memory and theory of mind). We used non-digital, manipulative material (pencil, paper, objects like dolls, cars, balls, among others; see Supplementary Table 1 for a detailed description of each test). These children were subdivided into seven well-balanced age groups, with the following number of children per group: 30–35 months: *n* = 98 (54 boys, 46 girls); 36–41 months: *n* = 97 (46 boys, 51 girls); 42–47 months: *n* = 102 (50 boys, 52 girls); 48–53 months: *n* = 101 (48 boys, 53 girls); 54–59 months: *n* = 87 (42 boys, 45 girls); 60–65 months: *n* = 83 (41 boys, 42 girls); 66–71 months: *n* = 75 (36 boys, 39 girls). We used a parent’s clinical questionnaire, adapted from the *Evaluación Neuropsicológica Infantil*^55^ to ascertain the presence of certain guidelines of typical development: (a) born at term (≥ 37 weeks of gestation)^56^, (b) birth weight between 2500 and 4000 g (5.5–8.8 lbs.)^57^, and (c) no reports of prenatal, perinatal or postnatal complications or trauma that could affect nervous system development^54,58^. No children had any diagnosis of neurological or psychiatric disorders. Trained psychologists assessed the children and interviewed the parents in well-lit locations, free of distractions. Rooms were provided by the day-care or preschool centers, but the children who were not in day-care or preschool were evaluated in their homes. All testing was done individually, within a single week, and lasted approximately 2 h, divided into three sessions of 40–45 min. each. The order of tasks presentation was counterbalanced. We explained the whole procedure, the use, and confidentiality of data, and provided feedback to the parents of the children when requested. Examiners were sensitive to the regional differences in spoken Spanish when testing children from different regions/countries^59^. Validation of these tests (consistency, reliability) was previously demonstrated^60–62^. All children were monolingual native Spanish speakers. Agreements were reached with each public and private institution to assess the children and collect the data; then, parents signed informed consent before testing their children. The ethics committee of our institution approved the tests we performed in this study, which followed the principles of the Helsinki Declaration (#ET062009-62; Instituto de Neurociencias, Universidad de Guadalajara).

### Analysis of neuropsychological functions

We normalized all the obtained scores to a scale 0–1 and grouped them into the five neuropsychological functions described above (receptive, expressive, attention/memory, processing, and executive functions). Due to our balanced group numbers, we did not consider size differences across age groups^12^. We calculated the peak global cross-correlations across tasks using the scores from either the entire data set or from subjects belonging to each age group. We estimated the differences in average correlation by subtracting the average correlation values from contiguous age groups.

### Cluster analysis

We partitioned the original data set into a discrete number of clusters by using the squared Euclidean distance and the k-means algorithm for cluster center initialization. We selected the optimal number of clusters by using the Calinski–Harabasz and the Davies–Bouldin criteria. We measured the absolute Euclidean distance between cluster centroids and the within-cluster sums of point-to-centroid distances as a function of age. From the original dataset, the scores from 32 children were partial and, therefore, could not be classified appropriately. We discarded these from subsequent analyses. We created cross-correlograms using data from subjects belonging to each cluster for each age group, and the average correlation difference was calculated between clusters for each age group. The average performance (and choice variance) from subjects belonging to each cluster for all ages or the different age groups was calculated and sorted either by neuropsychological function or by the summed correlations across tests (for the entire dataset). The polynomial coefficients (i.e., slopes and intercepts) from linear fits were obtained using a conventional least-squares algorithm.

We used measures from complex network analysis to characterize the relationship among tests^22^. In particular, we used a sensitive measure of global connectivity termed betweenness centrality (BC;^63,64^. This binary metric (using a cross-correlation threshold of 0.5) corresponds to the number of shortest paths that pass through each node. Consequently, ‘bridging’ nodes that connect separate parts of the network tend to have high BC. We calculated the summed BC across age groups and used a variable threshold in the summed BC to identify relevant tasks. Next, we used supervised machine learning tools to classify the children as belonging to each of the subclusters. The classification model was based on a k-nearest neighbor (KNN) classifier. We performed a tenfold cross validation and extracted the cross-validation loss of the predictive model. We also calculated the classification efficiency for the KNN-classifier as a function of the summed BC criterion described previously.

### Analysis of contextual factors that influence neuropsychological classification

We explored whether domestic and educational environments of the children could predict their classification cluster. We used a multivariable linear regression model (MLRM) to quantify the regression coefficients for these predictors, as described before^65^.

### Statistical analysis

All statistical tests were performed using MATLAB R2016a (MathWorks, Inc.; Natick, USA). For group comparisons, we used Kruskall-Wallis multi-comparison tests and repeated measures ANOVA (RM-ANOVA) tests, with Bonferroni’s or Wilcoxon Signed Rank *posthoc* tests. We employed nonparametric tests whenever the assumptions required to use the parametric versions were not met. Normality tests were performed using Lilliefors, and Jarque–Bera tests^66^. To quantify the significance of the MLRM coefficients, we contrasted the actual regression coefficients against 1000 surrogate data sets generated by shuffling the predictors, as previously described^65,67^. With this approach, we established the empirical significance of the observed coefficients by comparing them against coefficients obtained with randomly permuted predictors (i.e., the surrogates). In other words, we tested the null hypothesis that the regression coefficients were generated by chance. We illustrate our group data as averages ± S.E.M. with a significance set at *P *≤ 0.05. The number of children used for each analysis is represented inside parentheses in the corresponding figure panels.

## Supplementary Information

Supplementary Information.
