# Author Correction: Clustering of neuropsychological traits of preschoolers

**DOI:** 10.1038/s41598-021-91785-0

**Published:** 2021-06-08

**Authors:** Mario Treviño, Beatriz Beltrán‑Navarro, Ricardo Medina‑Coss y León, Esmeralda Matute

**Affiliations:** 1 Laboratorio de Neuropsicología y Neurolingüística, Instituto de Neurociencias, Universidad de Guadalajara, Guadalajara, México; 2 Laboratorio de Plasticidad Cortical y Aprendizaje Perceptual, Instituto de Neurociencias, Universidad de Guadalajara, Guadalajara, Jalisco México; 3 Departamento de Neurociencias, Centro Universitario de Ciencias de La Salud, Universidad de Guadalajara, Guadalajara, Jalisco México

Correction to: *Scientific Reports* 10.1038/s41598-021-85891-2, published online 22 March 2021

The Acknowledgements section in the original version of this Article was incomplete.

“This work was partially supported by a PROMEP grant (UDGPTC1217) awarded to BBN. RM-C received a M.Sc. scholarship from CONACYT. We gratefully acknowledge Dr. Mónica Rosselli and Dr. Alfredo Ardila for their invaluable contribution in all stages of the design, data collection and standardization of the ENI-P Battery. Thanks to Liliana Castillejo (Ph.D), Claudia Delfín (Ph.D), Paola López García (Msc), Maria Elena Navarro Calvillo (Ph.D), Yunuén Zalapa Medina (Ph.D) and Hugo Guadalupe Canto Pech (Ph.D) who supervised the data collection from Mexican children. Thanks to Nadyezhda Van Tuylen (Msc) who supervised the Guatemalan team, whereas Olber Eduardo Arango Tobón (Msc), and Martha Lucia Miranda Giraldo (Msc) coordinated the work in Colombia. Additionally, we would like to thank the institutions, children and families who contributed and participated in this study. We are grateful for the constructive feedback from the reviewers.”

now reads:

“This work was partially supported by CONACyT Ciencia Básica 2015-01 #251760, and by a PROMEP grant (UDGPTC1217) awarded to BBN. RM-C received a M.Sc. scholarship from CONACYT. We gratefully acknowledge Dr. Mónica Rosselli and Dr. Alfredo Ardila for their invaluable contribution in all stages of the design, data collection and standardization of the ENI-P Battery. Thanks to Liliana Castillejo (Ph.D), Claudia Delfín (Ph.D), Paola López García (Msc), Maria Elena Navarro Calvillo (Ph.D), Yunuén Zalapa Medina (Ph.D) and Hugo Guadalupe Canto Pech (Ph.D) who supervised the data collection from Mexican children. Thanks to Nadyezhda Van Tuylen (Msc) who supervised the Guatemalan team, whereas Olber Eduardo Arango Tobón (Msc), and Martha Lucia Miranda Giraldo (Msc) coordinated the work in Colombia. Additionally, we would like to thank the institutions, children and families who contributed and participated in this study. We are grateful for the constructive feedback from the reviewers.”

The original Article has been corrected.

