# Supplementary material for: Clustering of neuropsychological traits of preschoolers
Source: Sci Rep. 2021 Mar 22;11:6533. doi: 10.1038/s41598-021-85891-2 (PMC7985492; doi:10.1038/s41598-021-85891-2)
Supplement: Supplementary file 1 — Supplementary Information. [file 41598_2021_85891_MOESM1_ESM.pdf]

Supplementary Information:

Clustering of neuropsychological traits of preschoolers

Mario Treviño[2*], Beatriz Beltrán-Navarro[3], Ricardo Medina-Coss y León[2], Esmeralda Matute[1*]

[1]Laboratorio de Neuropsicología y Neurolingüística, Instituto de Neurociencias, Universidad de Guadalajara, Guadalajara, México, [2]Laboratorio de Plasticidad Cortical y Aprendizaje Perceptual, Instituto de Neurociencias, Universidad de Guadalajara, Guadalajara, Jalisco, México, [3]Departamento de Neurociencias, Centro Universitario de Ciencias de la Salud, Universidad de Guadalajara, Guadalajara, Jalisco, México.

*** Correspondence:**

Correspondence and requests for materials should be addressed to E.M. (email: ematute@ cencar.udg.mx) or M.T. (email: mariomtv@hotmail.com)

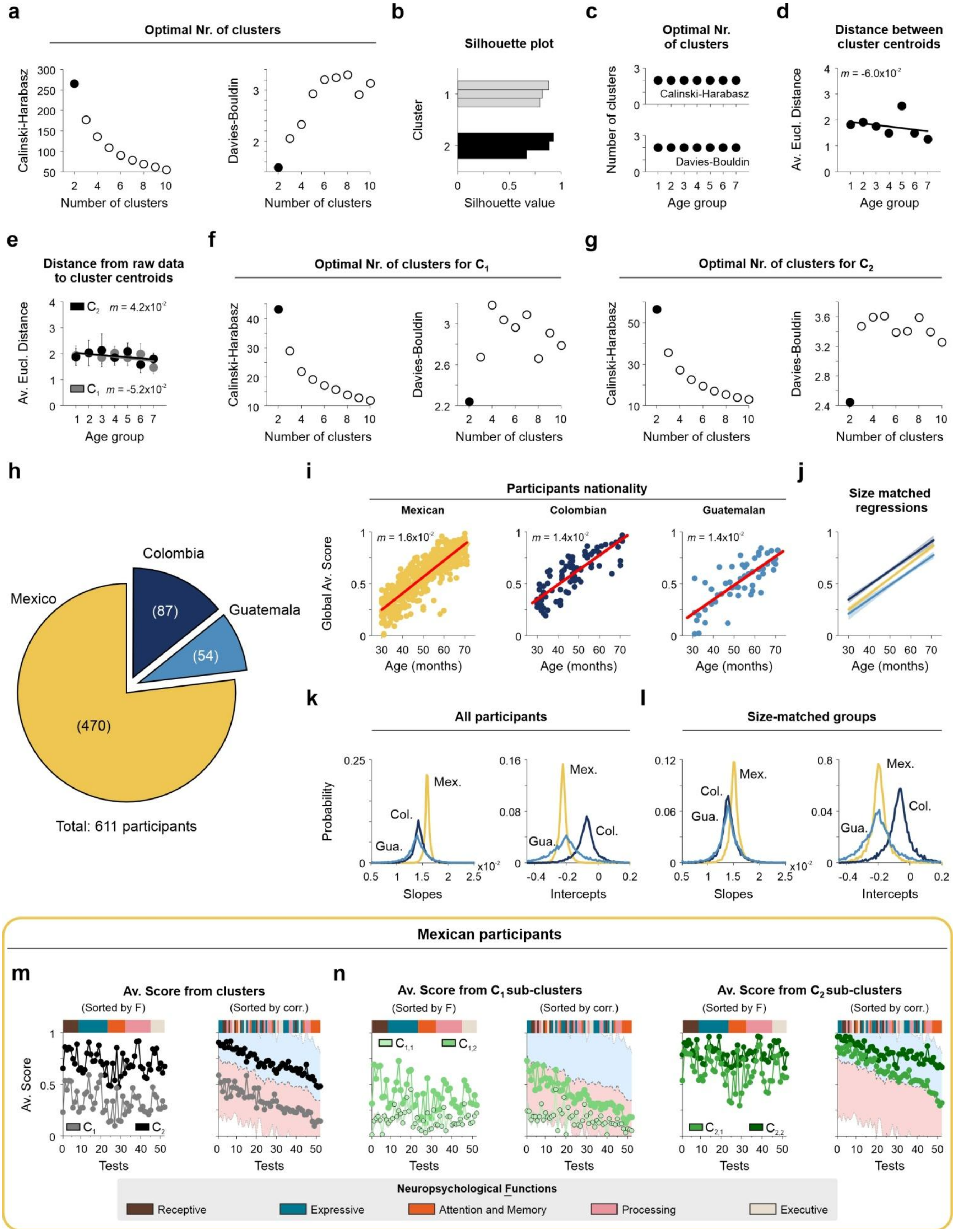
a
Optimal Nr. of clusters
Calinski-Harabasz
Number of clusters
Davies-Bouldin
Number of clusters
b
Silhouette plot
Cluster
Silhouette value
c
Optimal Nr. of clusters
Number of clusters
Calinski-Harabasz
Davies-Bouldin
Age group
d
Distance between cluster centroids
m = -6.0x10-2
Av. Eucl. Distance
Age group
e
Distance from raw data to cluster centroids
C2 m = 4.2x10-2
C1 m = -5.2x10-2
Av. Eucl. Distance
Age group
f
Optimal Nr. of clusters for C1
Calinski-Harabasz
Davies-Bouldin
Number of clusters
g
Optimal Nr. of clusters for C2
Calinski-Harabasz
Davies-Bouldin
Number of clusters
h
Colombia
Mexico
Guatemala
(87)
(54)
(470)
Total: 611 participants
i
Participants nationality
Mexican
Colombian
Guatemalan
Global Av. Score
m = 1.6x10-2
m = 1.4x10-2
m = 1.4x10-2
Age (months)
j
Size matched regressions
Age (months)
k
All participants
Probability
Mex.
Col.
Gua.
Slopes
Intercepts
l
Size-matched groups
Mex.
Col.
Gua.
Slopes
Intercepts
Mexican participants
m
Av. Score from clusters
(Sorted by F)
(Sorted by corr.)
Av. Score
C1
C2
Tests
n
Av. Score from C1 sub-clusters
(Sorted by F)
(Sorted by corr.)
C1,1
C1,2
Tests
Av. Score from C2 sub-clusters
(Sorted by F)
(Sorted by corr.)
C2,1
C2,2
Tests
Neuropsychological Functions
Receptive
Expressive
Attention and Memory
Processing
Executive

Supplementary Fig. 1 Classification of neuropsychological scores from Latin-American children. a K-means clustering evaluation for the results from the neuropsychological tests performed on 643 Latin-American children using the Calinski-Harabaz and Davies-Bouldin criteria. **b** Silhouette plot from the clustered data (using the two main clusters). **c** Optimal number of clusters using Calinski-Harabaz (upper panel) and Davies-Bouldin (lower panel) criteria from the scores of children with an age of 30-35 (group 1), 36-41 (group 2), 42-47 (group 3), 48-53 (group 4), 54-59 (group 5), 60-65 (group 6), or 66-71 (group 7) months. **d** Average Euclidean distance as the distance metric between cluster centroids as a function of age group. **e** Average distance from individual scores to cluster centroids as a function of age. Clustering evaluation for sub-clusters C_1 **f** and C_2 **g** using the Calinski-Harabaz and Davies-Bouldin criteria. **h** Pie chart representing the number of Mexican (primrose yellow, n = 470), Colombian (steel blue, n = 87), and Guatemalan (bonnie blue, n = 54) children participating in this study. **i** Global scores (average of all tests/participant) as a function of age for each nationality. **j** The linear fits (average ± S.E.M.) extracted by sub-sampling data. **k** Slope (left panel) and intercept (right panel) distributions for each nationality using sub-sampled data from all participants or **l** using data from size-matched groups. The clustering analysis applied to Mexican children only (*i.e.*, the largest group) confirms the existence of two main clusters **m** with two sub-divisions each **n**. The average performance from the children belonging to each sub-cluster reveals a grouping scheme that spans, coherently, from low performers to high performers. Data in panels **m-n** are grouped by neuropsychological function (left panel) and by summed correlation across tasks (right panels), respectively. Number of participants in parentheses.

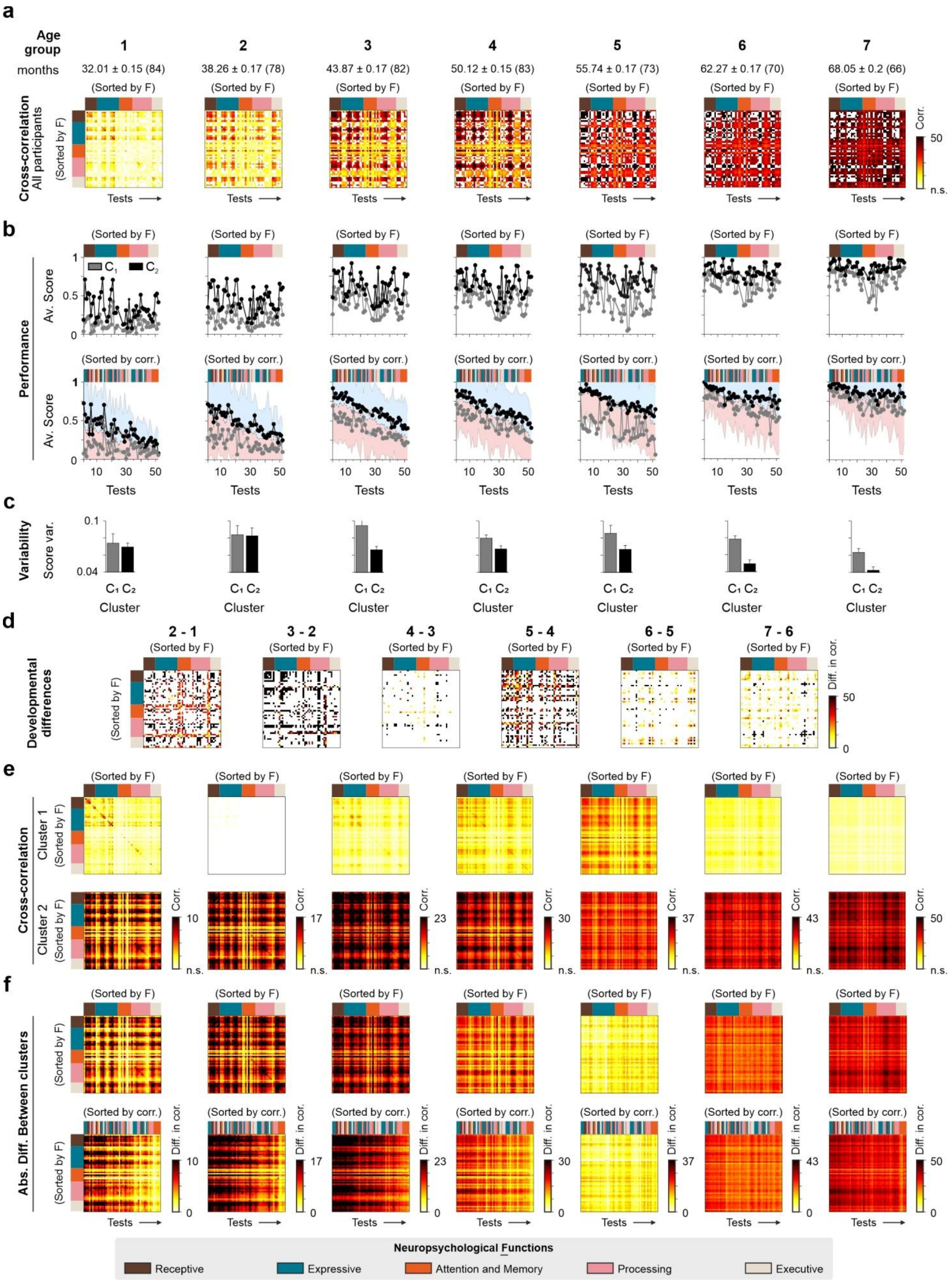
a
Age group
1
2
3
4
5
6
7
months
32.01 ± 0.15 (84)
38.26 ± 0.17 (78)
43.87 ± 0.17 (82)
50.12 ± 0.15 (83)
55.74 ± 0.17 (73)
62.27 ± 0.17 (70)
68.05 ± 0.2 (66)
(Sorted by F)
Cross-correlation All participants
Corr.
50
n.s.
Tests
b
Performance
Av. Score
C1
C2
(Sorted by corr.)
Tests
c
Variability
Score var.
0.1
0.04
C1 C2
Cluster
d
Developmental differences
2 - 1
3 - 2
4 - 3
5 - 4
6 - 5
7 - 6
Diff. in cor.
e
Cross-correlation
Cluster 1
Cluster 2
10
17
23
30
37
43
50
f
Abs. Diff. Between clusters
Neuropsychological Functions
Receptive
Expressive
Attention and Memory
Processing
Executive

Supplementary Fig. 2 Neurodevelopmental profile of cross-correlations across tasks. a Peak cross-correlations across tasks from children of the different age groups (average age in months and number of children displayed on top of each correlogram). **b** Average scores and **c** score variances of children classified into the two main clusters (C_1: gray and C_2: black) for each age group (columns). The first three age groups (1-3) presented differences in their average scores (Kruskall-Wallis test with Bonferroni *posthoc* correction, $P < 0.05$), whereas groups 4-7 did not ($P \geq 0.05$). Clusters from age groups 3,4,6,7 had different score variances (KW-test, $P < 0.05$). **d** Difference in peak cross-correlations across tasks from children from contiguous age groups. Filled colored pixels indicate significant correlations ($P < 0.05$). **e** Cross-correlations across tasks for each of the two main clusters as a function of age group (columns). **f** Absolute difference in cross-correlations between clusters 1 and 2. Inverted heat color bars with darker colors represent higher correlation values.

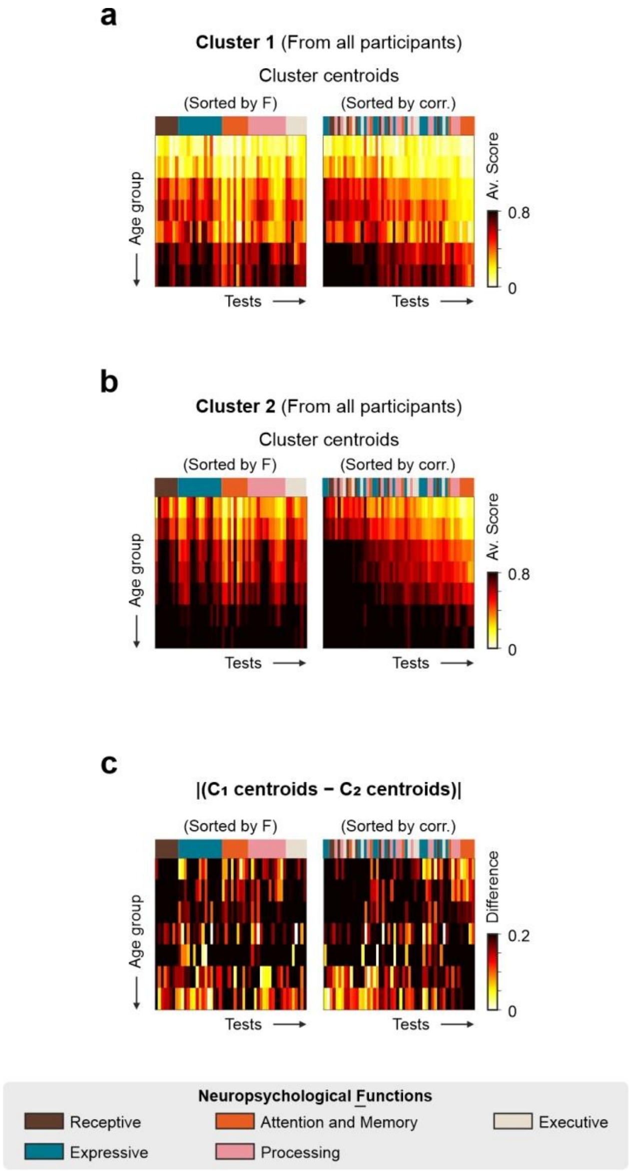

Supplementary Fig. 3 Cluster centroids from the different age groups. Cluster centroids as a function of age group (in rows) sorted by neuropsychological function (left panel) or by the summed correlations across tasks (right panel) for cluster 1 **a** and cluster 2 **b**. **c** Absolute difference between C_1 and C_2 centroids (KW-test, $P < 0.0001$ for all cases).

Neuropsychological Functions

Test #	Receptive functions	Description
	**Visual perception**	**Children are asked:**
1	Images	To match two images that are similar, to find the objects in an overlapping illustration, and to name the image in the visual closure task.
	**Auditory perception**	
2	Object-sound	To match sounds with their noisemaker.
	**Haptic perception**	
3	Object-Object	To decide if two objects are the same by touching them out of view.
4	Right hand recognition	To matchout of view objects by touching them with their right hand (one out of three possibile objects).
5	Left hand recognition	To matchout of view objects by touching them with their left hand (one out of three possibile objects).
	**Receptive Language**	
6	Comprehension	To answer questions about a story immediately after the examiner read it out loud to the child.
7	Instructions	To follow oral commands that entaill first using three objects, and later nine figures in an illustration.
8	Designation	To point out different body parts in a doll figure drawn on a chart or into themselves.

Test #	Expressive functions	Description
	**Fine Motor**	**Children are asked:**
9	Pegs preferred hand	To insert pegs in a pegboard with their preferred hand during 20 seconds.
10	Pegs secondary hand	To insert pegs in a pegboard with their secondary hand during 20 seconds.
11	Pegs both hands	To insert pegs in a pegboard using both hands during 20 seconds.
12	Board	To insert a peg in a target hole by manipulating a ring using two strings, one with each hand.
	**Ideomotor praxis**	
13	Facial praxis	To carry out, on command or by imitation, movements with different parts of the face, and facial gestures.
14	Limb praxis	To carry out, on command or by imitation, learned motor acts with their hand.
	**Constructional praxis**	
15	Blocks	To build three-dimensional designs with blocks.
	**Graphic skills**	
16	Human figure drawing	To draw a human figure.
	**Gross motor**	
17	Motor skills: Legs	To carry out, on command or by imitation, different movements with their legs.
18	Motor skills: Arms	To catch and throw a seed bag.
	**Expressive language**	
19	Personal information	To say their name and age.
20	Play	To perform pretended play with dolls and objects.
21	Naming	To name the body parts that the examiner points out, one-by-one, on a doll image or on to the child.
22	Fluency	To say as many animals as possible during one minute.
23	Repetition	To repeat sentences that the examiner says, one-by-one, in an increasing size order.

Test #	Attention and Memory	Description
	**Memory (coding)**	**Children are asked:**
24	Narrative memory	To re-tell a story immediately after it is read out loud by the examiner.
25	Visual sequence	To point out the same pictures, and in the same order, as the examiner does from a chart with nine pictures, increasing the amount of pictures per sequences.
26	Verbal sequence	To repeat a sequence of words, and in the same order, that the examiner said out loud, increasing the amount of words per sequence.
	**Memory (recall)**	
27	Differed narrative memory	To re-tell the same story from test #24, 20 minutes after the test was given.
28	Differed visual sequence	To point out the same sequence of pictures from the test #25, 20 minutes after the test was given.
29	Differed verbal sequence	To repeat the same list of words from test #26, 20 minutes after the test was given.
	**Attention**	
30	Word attention	To clap their hands every time that they hear the word "kiss" from a list of 25 word thay appeared randomly.
31	Cross-out	To cross-out all the bears contained in a work sheet.
32	Rhythm	To clap hands immediately after the examiner does, following a rhythm.

Test #	Processing functions	Description
	**Reasoning**	**Children are asked:**
33	Spatial terms	To follow oral commands that entail spatial words.
34	Stars	To place star stickers in a squares matrix following a given model.
35	Contrasts	To complete sentences with an antonym.
36	Classification	To classify geometrical figures by shape or color.
	**Mathematical skills**	
37	Estimation	To follow oral commands that entail quantity terms.
38	Counting	To count and say out loud how many chickens appear on an illustration (Max. 10 chickens)
39	Subitization	To say, as fast as possible, how many objects are in the cards, one-by-one, that are presented to them. Time is recorded.
40	Calculation	To answer two arithmetical problems.
	**Pre-reading skills**	
41	Syllables	To clap their hands according to the number of syllables of each word presented in an illustration.
42	Rhyme	To detect one word, out of three, which's final sound is different from the others.
43	Initial sound	To detect one word, out of three, which's initial sound is different from the others.
44	Name writing	To write their name.
45	Rapid naming	To name, as fast as possible, four objects that are repeated in a chart of 30 items.

Test #	Executive functions	Description
	**Cognitive flexibility / Shifting**	**Children are asked:**
46	Interference	To relate two objects in an opposite way to the usually done (reverse role game).
47	Categorization	To categorize cards following two different criteria and then to switch them.
	**Self-regulation**	
48	Gift	To keep turned back while the examiner wraps a present for them. Timed is recorded.
49	Statue	To keep still while standing. Time is recorded.
50	Sitting statue	To keep still while sitting. Time is recorded.
	**Working memory**	
51	Pointing-out	To point out different pictures from a series of cards with 2 to 8 pictures each.
	**Theory of mind**	
52	Beliefs	To express desires, knowledge out of context.

Data from Figure 1a

Supplementary Table 1 Neuropsychological tests. List of the 52 tests sorted by neuropsychological function.

Age group	Age range (Y:M)		Average age (Months)	Girls	Boys	Total
1	02:06	02:11	32.01 ± 0.15	46	52	98
2	03:00	03:05	38.26 ± 0.17	51	46	97
3	03:06	03:11	43.87 ± 0.17	52	50	102
4	04:00	04:05	50.12 ± 0.15	53	48	101
5	04:06	04:11	55.74 ± 0.17	45	42	87
6	05:00	05:05	62.27 ± 0.17	42	41	83
7	05:06	05:11	68.05 ± 0.20	39	36	75
			**Total:**	328	315	643

Data from Figure 1a

Supplementary Table 2 Number of girls and boys from the different age groups. This table corresponds to the data we illustrate in **Fig. 1a**.

a

Global Av. Score

Age Group	Av. Score (Probabilities) Cluster 1	Av. Score (Probabilities) Cluster 2
1	0.14 ± 0.02	0.35 ± 0.01
2	0.25 ± 0.03	0.47 ± 0.02
3	0.35 ± 0.05	0.61 ± 0.01
4	0.47 ± 0.02	0.63 ± 0.01
5	0.55 ± 0.04	0.75 ± 0.01
6	0.61 ± 0.03	0.79 ± 0.01
7	0.72 ± 0.01	0.85 ± 0.01
All ages	0.34 ± 0.01	0.71 ± 0.00

Data from Figure 3a

b

Type of school

Age Group	Public (Probabilities)		Private (Probabilities)		No school (Probabilities)	
	Cluster 1	Cluster 2	Cluster 1	Cluster 2	Cluster 1	Cluster 2
1	0.25 ± 0.03	0.24 ± 0.03	0.08 ± 0.02	0.27 ± 0.03	0.10 ± 0.02	0.06 ± 0.01
2	0.23 ± 0.04	0.21 ± 0.04	0.10 ± 0.03	0.27 ± 0.03	0.12 ± 0.02	0.06 ± 0.02
3	0.19 ± 0.04	0.32 ± 0.04	0.11 ± 0.03	0.28 ± 0.03	0.09 ± 0.02	0.02 ± 0.01
4	0.31 ± 0.04	0.34 ± 0.04	0.07 ± 0.03	0.25 ± 0.03	0.01 ± 0.01	0.01 ± 0.01
5	0.27 ± 0.04	0.35 ± 0.05	0.06 ± 0.03	0.30 ± 0.03	0.01 ± 0.01	0.01 ± 0.01
6	0.27 ± 0.05	0.36 ± 0.05	0.05 ± 0.03	0.32 ± 0.03	0.00 ± 0.00	0.00 ± 0.00
7	0.28 ± 0.04	0.30 ± 0.04	0.08 ± 0.02	0.32 ± 0.03	0.00 ± 0.00	0.01 ± 0.01
All ages	0.24 ± 0.01	0.34 ± 0.01	0.11 ± 0.01	0.24 ± 0.01	0.06 ± 0.00	0.01 ± 0.00

Data from Figure 3b

c

Household

Age Group	Father + Mother (Probabilities)		Mother (Probabilities)		Mother + Step Father (Probabilities)	
	Cluster 1	Cluster 2	Cluster 1	Cluster 2	Cluster 1	Cluster 2
1	0.34 ± 0.05	0.49 ± 0.05	0.08 ± 0.02	0.05 ± 0.01	0.00 ± 0.00	0.02 ± 0.01
2	0.38 ± 0.07	0.49 ± 0.07	0.07 ± 0.01	0.06 ± 0.01	0.00 ± 0.00	0.00 ± 0.00
3	0.32 ± 0.05	0.51 ± 0.05	0.06 ± 0.02	0.08 ± 0.02	0.00 ± 0.00	0.02 ± 0.01
4	0.30 ± 0.05	0.47 ± 0.05	0.08 ± 0.02	0.11 ± 0.02	0.00 ± 0.00	0.02 ± 0.01
5	0.26 ± 0.05	0.52 ± 0.05	0.08 ± 0.02	0.14 ± 0.02	0.00 ± 0.00	0.00 ± 0.00
6	0.27 ± 0.05	0.52 ± 0.05	0.05 ± 0.02	0.14 ± 0.02	0.00 ± 0.00	0.01 ± 0.01
7	0.29 ± 0.04	0.56 ± 0.04	0.03 ± 0.01	0.03 ± 0.01	0.03 ± 0.01	0.05 ± 0.01
All ages	0.34 ± 0.01	0.49 ± 0.01	0.06 ± 0.00	0.08 ± 0.01	0.01 ± 0.00	0.02 ± 0.00

Data from Figure 3c

Supplementary Table 3 Impact of domestic and educational factors on the classification scheme. a Average score probabilities ± S.E.M. for children from cluster 1 (C_1) and cluster 2 (C_2) as a function of age group (rows). **b** Average probabilities ± S.E.M. as a function of age group, that children from C_1 and C_2 belonged to a public, private or no-school. **c** Average probabilities ± S.E.M. as a function of age group, that children from C_1 and C_2 lived with both parents, with the mother only, or with mother and step father. This table corresponds to the data we illustrate in **Fig. 3a-c**.

a

Cluster 1

	Range (Min)	Range (Max)	Average (Years)
Father's age	20	48	33.29 ± 0.15
Mother's age	18	52	30.60 ± 0.17
Father's educational level	3	27	13.84 ± 0.17
Mother's educational level	3	25	13.94 ± 0.15

Data from Figure 3d-e

Cluster 2

	Range (Min)	Range (Max)	Average (Years)
Father's age	22	68	35.57 ± 0.40
Mother's age	21	48	32.44 ± 0.32
Father's educational level	2	25	14.41 ± 0.24
Mother's educational level	3	26	14.90 ± 0.21

Data from Figure 3d-e

b

Parents age

Age Group	Father (Years)		Mother (Years)	
	Cluster 1	Cluster 2	Cluster 1	Cluster 2
1	32.85 ± 0.58	35.24 ± 0.58	30.50 ± 0.49	32.24 ± 0.50
2	29.77 ± 1.04	32.88 ± 0.61	29.05 ± 0.60	30.74 ± 0.52
3	32.66 ± 0.69	35.18 ± 0.58	29.25 ± 0.52	31.48 ± 0.46
4	33.87 ± 0.73	35.28 ± 0.74	31.49 ± 0.74	32.58 ± 0.53
5	35.57 ± 1.09	37.36 ± 0.64	31.25 ± 0.95	33.05 ± 0.48
6	31.73 ± 1.24	34.58 ± 0.79	28.85 ± 1.05	31.82 ± 0.67
7	36.51 ± 0.89	35.66 ± 0.48	32.91 ± 0.84	33.18 ± 0.45
All ages	33.12 ± 0.22	35.51 ± 0.18	30.56 ± 0.19	32.44 ± 0.14

Data from Figure 3d

c

Educational level of parents

Age Group	Father (Years)		Mother (Years)	
	Cluster 1	Cluster 2	Cluster 1	Cluster 2
1	13.89 ± 0.43	15.88 ± 0.35	14.14 ± 0.41	15.95 ± 0.35
2	12.74 ± 0.61	15.08 ± 0.43	13.31 ± 0.55	15.48 ± 0.29
3	13.69 ± 0.64	14.67 ± 0.32	13.67 ± 0.60	15.44 ± 0.34
4	11.71 ± 0.73	14.56 ± 0.43	12.04 ± 0.62	14.83 ± 0.40
5	11.78 ± 0.73	15.19 ± 0.43	12.25 ± 0.73	15.54 ± 0.34
6	11.11 ± 0.60	14.49 ± 0.38	10.84 ± 0.65	15.13 ± 0.41
7	11.83 ± 0.58	14.72 ± 0.41	13.02 ± 0.48	14.56 ± 0.33
All ages	13.69 ± 0.15	14.42 ± 0.11	13.95 ± 0.13	14.84 ± 0.10

Data from Figure 3e

Supplementary Table 4 Impact of the educational level of parents on the classification scheme. a Average age (mean ± S.E.M.) of parents from children from cluster 1 (C_1) and cluster 2 (C_2). **b** Average age (mean ± S.E.M.) of parents of children from C_1 and C_2 as a function of children's age group. **c** Average years of schooling (mean ± S.E.M.) form parents of children from C_1 and C_2 as a function of children's age group. This table corresponds to the data we illustrate in **Fig. 3d-e**.
